# Valued attributes of professional support for people who repeatedly self‐harm: A systematic review and meta‐synthesis of first‐hand accounts

**DOI:** 10.1111/inm.12969

**Published:** 2022-01-15

**Authors:** Cara Sass, Cathy Brennan, Kate Farley, Helen Crosby, Rocio Rodriguez Lopez, Daniel Romeu, Elizabeth Mitchell, Allan House, Else Guthrie

**Affiliations:** ^1^ Leeds Institute of Health Sciences School of Medicine University of Leeds Leeds UK; ^2^ Leeds Trinity University Leeds UK; ^3^ Leeds and York Partnership Foundation Trust Leeds UK; ^4^ School of Psychology University of Leeds Leeds UK

**Keywords:** psychotherapy, qualitative research, self‐harm, self‐injurious behaviour, systematic review, therapeutics

## Abstract

Therapeutic interventions are an important adjunct to self‐help strategies for people who self‐harm. There is little guidance for those offering therapy on the effective components of interventions for people who self‐harm. This was a systematic review aiming to identify the factors that contribute to positive experiences of therapy as described by people who have reduced or stopped self‐harm. The review followed PRISMA guidelines to locate and synthesize peer‐reviewed qualitative studies describing experiences of therapy among people who had reduced or stopped self‐harm. Study selection, data extraction, and quality assessment were peer reviewed and conducted for at least two researchers independently. Relevant first‐hand quotations were extracted from eligible studies and synthesized using a thematic analysis in collaboration with experts with personal and professional experience of self‐harm. Twenty‐three studies met eligibility criteria. Themes identified in the reported accounts were arranged under two meta‐themes. ‘Positive aspects of seeing a professional’ identified aspects of professional care that were common to all encounters: the value of sharing, space to talk and reflect, and the boundaries inherent in contact with a professional. ‘Positive attributes of individual professionals’ depended upon individual characteristics: the ability to build reciprocal trust by being non‐judgemental, showing genuine empathic concern, and being confident to talk about and respond directly to self‐harm. Our review indicates that therapeutic alliance is perceived as key to effective professional help for self‐harm, irrespective of underlying principles of therapy. All forms of therapy should be timely and reliable and centred around the needs of the individual and their experience of self‐harm.

## BACKGROUND

### Self‐harm as a public health concern

Self‐harm, a term used to describe an act of intentional self‐injury or poisoning (Hawton *et al*. 2003), is a public health concern owing to its direct consequences for health. This is especially true for repeated self‐harm, which is linked to increased risk of later suicide (Carroll *et al*. 2014). Suicidal intent is a part of the motive for some but not all acts of self‐harm, which can serve various functions for an individual, including: ‘responding to distressing thoughts or memories; responding to negative feelings about self – either by self‐punishment or by experiencing the cathartic or cleansing effect of the act; *managing powerful feelings* – typically either suicidal or aggressive in nature; *communicating distress*; *generating positive feelings* – which include a sense of warmth or relaxation, or powerfulness, or being in control, at times even a sense of arousal or excitement during the build‐up to an act (Edmondson *et al*. 2016).

Many people who self‐harm try to cope without the aid of professional services (Borges *et al*. 2010). Self‐help resources may be more acceptable than engaging with formal services due to concerns around availability, expected stigma, and confidentiality (Wadman *et al*. 2020). Self‐help is available in the form of websites providing advice and information, helplines, and peer‐led social networking groups; advice and support accessed through such platforms can be variable in terms of quality or adherence to current evidence (Romeu *et al*. 2020). Individuals may also seek alternatives to formal therapy because of perceived or actual negative experiences (Jones *et al*. 2011). Personal assets such as supportive social networks and aspirational goals (such as career success) may lead to a reduction or cessation of self‐harm (Brennan *et al*. in press).

However, self‐harm and suicidal thoughts are associated with social isolation (Dennis *et al*. 2007; Haw & Hawton 2011; Hawton & Harriss 2006) and disproportionately affect individuals with lower social or economic capital (Ayton *et al*. 2003; Iemmi *et al*. 2016; Mok *et al*. 2018) so it cannot be assumed that people who self‐harm will have good access to resources to help themselves.

Those who seek support for self‐harm may come into contact with a range of services: from general practice, emergency hospital departments, specialist mental health services, and to the voluntary sector (Warm *et al*. 2002). However, access to appropriate psychological services for people who self‐harm is limited with many offered little or no specialist aftercare (House & Owens 2020). Even when psychological services are available, they may not be tailored to, or cater for, people who self‐harm. For example, the largest psychological treatment service in England (the Improving Access to Psychological Services service (IAPT)) may not accept people who self‐harm if they pose immediate safety concerns (Saunders & Smith 2016).

Even when psychological therapies are available, professionals may be ill‐equipped to address self‐harm in their practice (Conlon & O’Tuathail 2012; Long & Jenkins 2010; McHale & Felton 2010). Professionals may hold negative attitudes to self‐harm, affecting the outcome of treatment and reinforcing feelings of stigma that may impede future help‐seeking behaviour (Karman *et al*. 2015; McHale & Felton 2010; Saunders *et al*. 2012; Timson *et al*. 2012). Few studies have explored the features of psychological therapy for self‐harm which are valued by recipients and considered to lead to positive outcomes.

The aim of this review is to identify the factors that contribute to positive experiences of therapy for people who self‐harm, taken from the direct accounts of people who have experienced psychological therapy and have reduced or stopped self‐harm.

## METHODS

We undertook a systematic review, namely, a review which utilized a search strategy and criteria for including/excluding studies, to identify all eligible articles and extract relevant first‐person accounts (Martinic *et al*. 2019). We applied qualitative meta‐synthesis approach to critically integrate the findings from multiple studies with a common focus to identify shared meanings and create novel thematic insights (Noblit & Hare 1988).

### Study identification

Commensurate with guidance for conducting systematic literature reviews (Martinic *et al*. 2019; Moher *et al*. 2015), we developed a strategy for locating relevant articles in collaboration with an information specialist. Search terms related to self‐harm or self‐injury were combined with terms related to reduction or cessation (i.e., ‘overdose’ or ‘burn’, and ‘abstain’, ‘protect’, etc.). We adapted our strategy based on the parameters of each database. We searched for articles published between 1947 and August 2019 in the following academic databases: MEDLINE, Embase, PsycINFO, CINAHL, Epistemonikos, Cochrane Database of Systematic Reviews, the Cochrane Central Register of Controlled Trials (CENTRAL), and the Web of Science (WOS): Citation Index and Conference Proceedings were also searched for grey literature. We utilized search engine citation alerts (Google and Web of Science), hand‐searched reference lists from relevant articles and literature reviews, and expert advisors working in self‐harm. A methodological filter was applied to the searches to retrieve qualitative research. This provided first‐hand accounts of the experience of seeking professional help after self‐harm. Members of the team completed a rigorous study screening and selection process according to PRISMA guidelines (Moher *et al*. 2009), adopting the inclusion/exclusion criteria shown in Table 1. An example of this search strategy in MEDLINE is available in the appendices; the full search strategy can be requested from the authors.

**Table 1:** Criteria for systematic review inclusion and exclusion

Inclusion Criteria	Exclusion Criteria
Studies that report first‐hand accounts associated with reduction or cessation of self‐harm from people who have self‐harmed. Studies of individuals of any age, gender, or ethnicity. Studies of individuals with or without co‐occurring psychiatric disorders. Studies across all motives (non‐suicidal or suicidal) and methods (poisoning or self‐injury) of self‐harm. Studies using a qualitative research design. Studies published in peer reviewed journals. Studies written in any language, providing an English‐language version is available.	Studies that focus solely on suicidal thinking or completed suicide with no reference to acts of self‐harm. Studies that report factors associated with a reduction or cessation of self‐harm but not first‐hand accounts. Studies that report only second‐hand accounts of how people have reduced or stopped self‐harm, e.g., healthcare professionals’ views towards self‐harm reduction or cessation. Studies that report only first‐hand accounts of self‐harm reduction or cessation with no reference to interactions with professionals

A full description of this process is provided elsewhere (Brennan *et al*., in press).

### Study selection

**Fig. 1:**
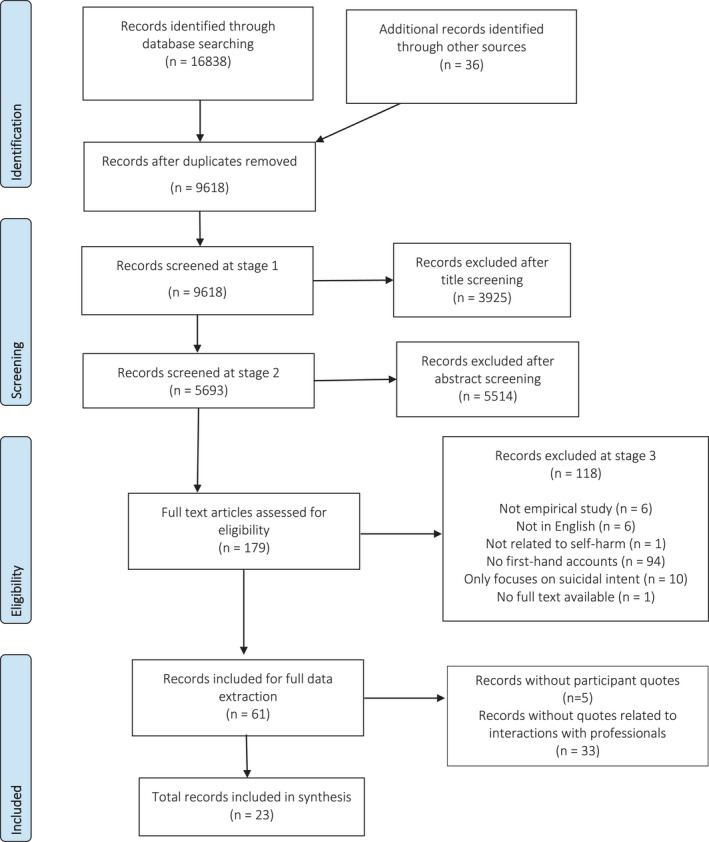
PRISMA diagram of study selection.

Relevant articles were collated and imported into a reference management database and screened for eligibility in three stages. An initial title screen filtered results to studies relevant to self‐harm, before applying inclusion criteria to a further title and abstract screen. The remaining articles were subjected to full manuscript screening to determine the full set of included articles. Two or more team members independently reviewed the inclusion/exclusions at each stage, and any discrepancies were discussed for consensus. Figure 1 provides an illustration of this process with quantities included/excluded during each step.

### Data extraction and quality assessment

Two reviewers extracted relevant contextual information (first author, year of publication, title, summary of participants, etc.) and data consisting of all qualitative accounts featured in the remaining manuscripts. These accounts took the form of interview extracts or questionnaire responses. Table 2 provides key information extracted from the included articles and quality ratings in this review.

**Table 2:** Reporting quality criteria (Carroll *et al*. 2012)

Criterion	Categorisation	Definition
The question and study design	Yes	If the choice of study design was given and explained
	No	If article does not specify question and study design
The selection of participants	Yes	If the selection of participants is described explicitly as, e.g. purposive, convenience, theoretical and so forth
	No	If only details of participants are given
Methods of data collection	Yes	If details of the data collection method are given, e.g. piloting, topic guides for interviews, number of items in a survey, use of open or closed items, validation, and so forth
	No	If just only states focus group, interview or questionnaire
Methods of analysis	Yes	If details of analysis method are given, e.g. transcription and form of analysis (with reference to or full description of method), validation tests, and so forth
	No	If only states content analysis or that data were analysed

Each study was given a quality assessment based on the adequacy of the reporting of the methods of study design, selection of participants, data collection, and data analysis. For each section where the reporting was adequate and appropriate for the study design, a score of 1 was given. Each study had a final score of 0–4. The criteria were based on established assessment tools: Consolidated criteria for Reporting Qualitative research (COREQ; Long & Godfrey 2004; Tong *et al*. 2007) and Strengthening the Reporting of Observational studies in Epidemiology (STROBE; Knottnerus & Tugwell 2008) and adapted as a single measure by Carroll *et al*. (2012). These criteria are given in Table 2.

Ratings did not influence the inclusion/exclusion process but were used to indicate the quality of each article in terms of research design and reporting. The extraction and quality assessment were carried out independently by at least two team members for each manuscript, with any conflicts discussed between reviewers to reach consensus.

### Data synthesis

Data were synthesized using a thematic process outlined by Thomas *et al*. (2017). This process was grounded in the data, involving development of a thematic framework using open coding of meanings within the set of first‐hand accounts, categorizing these accounts into influential ‘actions’ and helpful ‘mechanisms’. Related codes were grouped together into themes, which were then grouped into meta‐themes which had relative meaning.

The second phase of the synthesis involved a series of reflective group discussions with a reference panel of people with personal experience of self‐harm to refine this thematic framework. An initial thematic framework was presented with the associated quotations and the panel were asked to make comment and re‐arrange the quotation maps if warranted. Discussion centred on the interpretation of the quotations to ensure that the results remained rooted in the data extracted. A number of themes were altered and renamed in light of these discussions. Latent theme titles and definitions were then produced by the research team, using NVivo qualitative data management software (QSR 2021).

## RESULTS

The full search strategy returned 9626 studies excluding duplicates. Of these, 56 met the inclusion criteria after full‐text screening, and 23 articles contained direct accounts related to interactions with health professionals. These were published between 2003 and 2018, originating from Norway (*n* = 5), Canada (*n* = 5), UK (*n* = 5), USA (*n* = 3), Finland (*n* = 2), Sweden (*n* = 2), and The Netherlands (*n* = 1). The overall age range of participants in the review was 12–89 years. The majority of articles (*n* = 12) represented community samples; with inpatient (*n* = 4), combined inpatient/community (*n* = 3), online (*n* = 2), and prison populations (*n* = 1) also included. One article did not provide a description of the setting for recruitment. The numbers of participants ranged from 8 to 836, although most articles drew from a modest sample (mode across studies = 13). All but *n* = 2 articles utilized a larger sample of women than men. Extracted data for all included studies are given in table 3.

**Table 3:** Articles and key information extracted for the metasynthesis

First author + year	Study aim(s)	Method of data collection	Definition of self‐harm	Research setting	Form of professional support	Research location	Sample size	Age range (mean)	Gender split of participants (percentage)	Method(s) of self‐harm reported	Key findings	Quality rating
Bergmans *et al*. (2009)	Capture the experience of living in the ambivalent space between life and death for adults with recurrent suicide attempts (RSA).	Interviews	recurrent suicide attempts	Women with a history of self‐reported repeated suicide attempts (RSA) who had participated in a therapeutic intervention at the research site	PISA facilitated by clinicians (roles not specified)	Canada	8	Mean = 25.8	14 female (90%), 2 male (10%)	Not stated	1. Surviving moment to moment1.1 Deciding not to die in the moment1.1.1 Enduring life1.1.2 Seeing doom1.1.3 Turning the corner1.2 Deciding to live in the moment1.2.1 Gaining control1.2.2 Accepting help1.2.3 Making connections	4 (adequate)
Bergmans Gordon and Eynan (2017)	To develop a grounded theory of recovery from the perspective of young adults with a history of repeated suicide‐related behavior who completed at least one cycle of a specific treatment intervention for people with recurrent suicide attempts	Interviews	self‐identified history of two or more suicide attempts with intent to die	Young adults initially assessed for admission to the PISA intervention when they were 25 years old or younger.	Clinicians, roles not specified	Canada	16	30–60 years (43)	8 female (100%)	Not stated	1. A model of recovery1.1 Living to die1.2 Ambivalence and turning points1.3 Pockets of recovery	4 (adequate)
Borrill,(2005)	explores the subjective interpretations of precipitating events through interviews with survivors of potentially lethalattempts	Interviews	"Potentially lethal act of self‐harm"	Female prisoners	Psychiatrists; prison staff	England	15	19–50	women	Not stated	Very few references to anything helping. No thematic structures identified of relevance to this review.	3 (adequate)
Bostik (2007)	To understand how adolescents perceive attachment relationships as helping them overcome suicidality	Interviews	Being suicidal; defined as the report of suicidal ideation (persistent thoughts about intentionally ending one’s life) and/or suicidal behaviour (a purposeful act of self‐injury where the intent was death)	Participants were recruited through newspaper advertisements and brochures mailed to community service agencies	A counsellor	Canada	50	13–26 (21.9)	41 female, 9 male	Not stated	Attachment relationshipsexpereinces of attachmentchanging self‐perceptions	4 (adequate)
Chan *et al*. (2017)	To examine the recovery experience following a suicide attempt, and how narratives fitted into Lakeman and Fitzgerald's themes	Other: secondary analysis of narratives	Self‐reports: narrative descriptions of suicide attempts, self‐harm, risky behaviours and persistent suicidal ideation	Online ‐ anonymous submissions to a website	A therapist (general), trauma therapist	Online	113	N/K	N/K	Not stated	Suffering/psychache and struggle: abuse; devaluing self/family: disconnection/isolation/guilt/shame; loss; suicide ideationConnection: healthcare professionals; reconnecting with self; religion/spirituality; valuing family and friendsTurning point: hospitalization; realizing impact on family; things to be donesuicide and coping: goals for the future; healthy behviours; suicide as coping	2 (adequate)
Crona (2017)	To examine personal strategies to continue living after a suicide attempt	Interviews	"suicide attempters"	Participants had been admitted to the Department of Psychiatry, University Hospital, Lund, in 1956 up to 1969 and had received the diagnosis ‘severe depression/melancholia’ by a senior psychiatrist.	Specialist psychiatric staff; doctors, nurses	Sweden	13	62–89 years	N/K	Not stated	Findings presented under three main themes: 1. coming under professional care,2. experiencing relief in the personal situation,3. making a decision to continue living	3 (adequate)
Han (2015)	By shedding light on Korean‐Canadian immigrants’ help‐seeking for and self‐management of suicidal behaviours, offering guidance towards developing culturesensitive suicide prevention programs.	Interviews	"Suicidal behaviours, classified into three subsequent categories: 1) suicidal ideation; 2) suicide plan; and 3) suicide attempt"	First‐generation Korean‐Canadian immigrants who had experienced suicidal behaviours; recruited from community sample	Counsellors	Canada	15	20–62 (32.6)	11 women and 4 men	not stated nb ‐ only two of the participants had attempted suicide	Two overarching themes: resisting professional help; developing effective self‐management strategies	3 (adequate)
Holm (2011)	To explore how a recovery process facilitated changes in suicidal behaviour in a sample of women with BPD	Interviews	"suicidal behaviour ‐ an interpersonal event, displaying communication of intent in order to alleviate mental pain, that is, emotional pain and ‘psychache’"	Participants recruited via mental health nurses, therapists in different settings, and the ‘mental health’ organization on the west coast of Norway.The inclusion criterion was women diagnosed with BPD. All participants described histories of suicidal behaviour, and 11 continued suicidal behaviour in times of crisis.	Mental health nurses, therapists, professionals in hospital (role not specified)	Norway	13	25–53 (39)	13 Female (100%)	Not stated	Thematic structure: 1. Struggling to assume responsibility for self and others1.1 The desire to recover by searching for strength1.2 The struggle to be understood as the person you are2. Struggling to stay alive by enhancing self‐development2.1 Recovering by refusing to be violated2.2 Recovering by feeling safe and trusted	3 (adequate)
Holm (2010)	To explore and interpret women’s desire to survive emotional pain related to self‐harm.	Interviews	"intentional damage to one’s own body, apparently without a conscious intent to die"	Women living with borderline personality disorder (BPD), recruited by mental health clinicians from a variety of settings.	Psychiatric nurses	Norway	13	25–53 years (39)	13 female (100%)	Not stated	Identified thematic structure: 1. Self‐sacrifice*1.1 Self‐harm: A struggle to be relieved of responsibility*1.1.1 Desire to feel worthy in order to avoid self‐condeming feelings of guilt and shame1.1.2 Desire to become invisible in order to preserve one's self‐image*1.2 Fear of intimacy versus instrusion*1.2.1 Desire to be protected and to be cared for in times of crisis1.2.2 Searching for someone who understands one's fear of being alone and abandoned	3 (adequate)
Kool (2009)	To understand how the process of reducing or stopping self‐injury develops in patients with a history of severe self‐injury, and what factors play a role in that process?	Interviews	“Direct pain or injury inflicted by a person on his or her own body in a repeated pattern, usually with a low risk of fatality and without deliberate suicidal intent”	Participants with a history of severe self‐injurious behavior, attending a psychiatric intensive treatment center delivering specialised care for patients with behavioral problems triggered by psychiatric disorders.	Carers' and nurses at an intensive treatment centre	Netherlands	12	26–60 years (39)	12 female (100%)	Cutting; Burning/Branding (cigarettes, hot objects); Scratching/Picking; Banging the head; Squeezing; dropping oneself; hitting oneself; hitting with objects; biting; scolding; Breaking bones; punching; Inserting needles/sharp objects; starving; binding off toes; pulling off nails	Phase 1: Limit setting and connectingPhase 2: Self‐esteemPhase 3: Learning to understandPhase 4: AutonomyPhase 5: Stoping self‐injury and learning new strategiesPhase 6: Maintenance	4 (adequate)
McAndrew (2014)	To elicit the narratives of young people who engage in self‐harm and suicidal behaviour and identify what was helpful and/or unhelpful, and what their future needs might be from a diverse range of statutory and non‐statutory services.	Interviews	"Self‐harm (the deliberate destruction of one’s own body tissue with no suicidal intent) and suicidal behaviours (demonstrating suicidal intent)"	Young people who had experience of self‐harm and/or suicidal behaviour	Counsellors (school and CAMHS)	UK	7	13–17 years	7 female (100%)	Not stated	1. Cutting out the stress2. Stepping onto the path of help3. Cutting to the chase: prioritixing self‐harm on the public health agenda	1 (inadequate)
Perseius *et al*. 2003)	To investigate patients and therapists perception of receiving and giving dialectical behavioral therapy (DBT).	Interviews	"Suicide attempts and acts of deliberate self‐harm"	People with borderline personality disorder who self‐harm (not specified where they were rectuited), and active DBT therapists	DBT Therapists	Sweden	11	22–49 years (median 27)		Not stated	Only the patient categories reported (see paper for therapist categories)**1. The therapy effect**1.1 The therapy is life‐saving1.2 The therapy provides skills to help conquer suicidal and self‐harm impulses1.3 The therapy helps in accepting your feelings and not condemning (yourself or others)2. The effective components of the therapy and thearpists perception of working with DBT2.1 Respect and confirmation is the foundation2.2 The method of therapy brings understanding and focus on the problems2.3 Your own respondibility and the stubborn struggle with yourself2.4 The therapy contract brings support and challenge2.5 The group therapy‐hard but necessary2.6 The telephone coaching‐important crises support**3. Perceptions of psychiatric care before DBT**3.1 Not being understood and disrespectful3.2 Discontinuity and betrayal3.3 The poorly adapted tools of psychiatric care	2 (adequate)
Rissanen (2009)	To describe help from the view‐point of self‐mutilating Finnish adolescents	Interviews and written descriptions	"Self‐mutilating"	Finnish adolescents, who are or have self‐cut, responding to adverts in 4 magazines targeted at adolescents	Specialist and primary level professionals, school counsellors	Finland	72	13–18 years	319 girls (92%)28 boys (8%)	Self‐cutting	**1. Factors that enable help seeking‐**becoming conscious of being in need of help, knowledge of self‐mutillation as a phenomena, knowledge of available help for self‐mutilation, a caring environment, all kinds of support from friends, peers and parents **2.helpful factors** bening conscious of one's need for help, enabling early and practical intervention for all kinds of problems to adolescent, intervening in adolescents mutiliation, learning to discuss in general and expecially self‐mutilation and all kinds of emotions and difficult experiences with someone, authentic caring for the adolescent, adolescents own activities, either constructive or destructive.**3. Help Hindering Factors**	1 (inadequate)
Rissanen (2013)	To describe the factors contributing to the stopping of self‐cutting among 13–18‐year‐old Finnish adolescentsfrom the personal perspective of the adolescents	Questionnaires	Self‐cutting	Adolescents in comprehensive/upper secondary schools	Psychologist/psychiatrists delivering therapy; helpline staff; inpatient staff	Finland	347	12–21 years	10 girls interviewed gender split not recorded for 62 descriptions	Self‐cutting	**1. Factors associated with self‐cutting**Realising the uselessess of self‐cutting; Realising the irrationality of self‐cutting; Realising the stupidity of self‐cutting; The unhelpfulness of self‐cutting; The unattractiveness of self‐cutting**2. Personal factors**An improvement in one’s life situation; The negative physical sequels of self‐cutting: scars, prints, pain; The personal will to stop; Maturation; Improved mood; Being discovered or fear of it; The negative emotional sequels of self‐cutting; Faith; Not having enough strength to self‐cut; Unwillingness to hurt others; Flamenco dancing; Being able to release bad feelings in another way**3. The factors associated to other people**Existance of friends; Existance of a loved one; Support from others; Interfering self‐cutting by others; Discussing problems with others; Negative social consequences of self‐cutting; A promise to a significant other; Existance of siblings; Pointlessness of self‐cutting in relation to others**4. The factors associated with care or therapy**Therapy with a psychologist; Unspecified care or help; Therapy with a psychiatrist; Unspecified therapy; Inpatient care; Care from a school nurse**5. Unidentified factors** **6. Meanings related to the instruments used to cut**	4 (Adequate)
Shaw (2006)	To understand how women make a shift away from self‐injury and the role of professional treatment or the lack thereof in this process.	Interviews	“the deliberate, non‐life‐threatening, self effected bodily harm or disfigurement of a socially unacceptable nature"	All‐female college; students recruited through flyers posted at colleges in the US.	Psychologist/therapist, counsellor, professionals in unspecified roles from inpatient/outpatient services	USA	6	18–21	all women	Not stated	Professional treatment;motivators to stop/deterrentsrelational; ties and supportdesire/decision to stopelimination/decrease in psychological catalysts to self‐injurymeanings of self‐injury and problem identificationdisclosureself‐initiativemomentumlife committments and engagements	3 (adequate)
(Sinclair & Green 2005)	Explored how participants perceived the move away from deliberate self harm.	Interviews	"Standard clinical definition"	Long‐term follow up of previosuly described cohort	GPs, school counsellors,	UK	20	23–55 years	12 F, 8 M	Not stated	Resolution of adolescent chaosRecognition of alcohol as a factorSeeing deliberate self‐harm as a consequence of illness	4 (adequate)
Tofthagen (2017)	To explore, describe and understand former patients’ experiences of the process of recovery from self‐harm.	Interviews	Multiple forms reported	Two mental health organisations	Mental health nurses working in inpatient services	Norway	8	N/K	N/K	All had cut themselves repeatedly from moderatelyto more seriously over time and seven hadattempted suicide once or more. Seven had engaged in other forms of self‐harm such as overdosing, sticking sharp objects into the body, swallowing sharp objects, substance abuse, eating disorders and/or burning the skin Range 8–33 years of SH. At least 2 years no SH.	Main theme:‐ Recovery from self‐harm as an individual, prolonged learning processTheme 1 The turning point as the start of the transition process: Subthemes =To choose life; To verbally express one’s inner pain; To reconcile with one’s life historyTheme 2 Coping with everyday life ‐ an individual process: Subthemes =To choose other actions, in place of self‐harm; To attend to one’s basic, physicalneedsTheme 3 Valuing close relationships and relationships with mental health nurses‐ a social process. Subthemes = To receive support from close relationships; To receive guidance from mental health nurses	4 (adequate)
Vatne (2018)	To develop knowledge on what alleviates the suicidal suffering after having survived a suicide attempt.	Interviews	Suicide attempts not otherwise defined	Participants were invited to take part by a psychology specialist in connection with a follow‐up after suicide attempts	GPs; emergency department staff; mental health practitioners (nurses, psychiatrists, psychologists, therapist of unknown background)	Norway	10	21–52 years	4 F 6 M	Not stated	Three main themes: (1) experiencing hope through encounters,(2) experiencing hope through the atmosphere of wisdom(3) experiencing a ray of hope from taking back responsibility.	1 (inadequate)
Vatne (2016)	What resources in the person and their surroundings are crucial in a suicidal crisis to maintaining the will to live and hope for life?	Interviews	"Serious suicidality" after a suicide attempt	Hospital attendance following a suicide attempt	General professionals, GPs, crisis resolution teams, psychologists	Norway	10	21–52 years	4F 6 M	Not stated	Three main themes related to maintaining the will to live and hope in a period of crisis: Becoming aware of the desire to live; an experience of connectedness; someone who cares.	2 (adequate)
Weber (2002)	To describe how self‐abusing women in a locked, state psychiatric hospital defined self‐abuse in the context of their lives.	Interviews	"Self‐abuse"	Nine women living in a locked women’s unit in a large state psychiatric facility, who showed some type of self‐abusive behavior during their hospital stay or before admission	Nurses and non‐clinical staff	USA	7	21–48 (32) years	7 females (100%)	Not stated	Two overarching themes: **1. The reasons why** **2. What stops it**2.1 Talking to me2.2 Distraction2.3 Caring relationships2.4 Comfort2.5 Hope	3 (adequate)
Whitlock (2015)	To compare differences between past and current NSSI	Questionnaires (free text responses)	Participants self‐reported from a list of 19 behaviours	Students from 8 US colleges	Therapy through a phychiatrist	USA	836	mean 21.3 years	74% F 26%M	Self‐injury, various methods	Connection with others; Positive connectionsNegative effect on cared for othersRemoval of negative relationshipsProfessional/Therapeutic SupportEmotion RegulationSelf‐awarenessCoping skills (tools/behaviors or direct differences)Life circumstances changedFear of consequencesEnvironmental/SocialPhysical effectsMaturityMinimal life effects	4 (adequate)
Williams (2018)	To explore the perceptions of clinical services within self‐harm online communities, in particular: (1) their attitudes toward clinical services, (2) their reasons for choosing not to seek help and, (3) of the subset that do seek help, how their views of clinical services differ and what value they find in these services.	Other: Online forum threads	Self‐injury, or self‐poisoning, irrespective of suicidal intent	Users of three online communities for self‐harm	Health professionals, role not specified	Not specified; Online	209	Not stated	Not stated	Not stated	**1. Difficult to reach appropriate services in a timely manner**1.1 Inconsistent or limited access1.2 Unsure which services are available beyond GP1.3 Unsuitability of services**2. Access to therapy; through the medical gateway**2.1 Need for support to stop self‐harm (help‐seeking)2.2 Value of dealing with underlying issue**3. Confidentiality ‐ fear of disclosure and consequences**3.1 Not seeking physical healthcare due to disclosure3.2 Concerns of confidentiality3.3 Fear of labeling/being seen as mentally ill3.4 Avoidance responses**4. Value of support**4.1 Self‐care4.2 Support from online peers4.3 Encouragement of professional help	2 (adequate)
Wills (2013)	To explore the meaning of recovery from the perspectives of 6 individuals who self‐injure	Interviews	"Direct pain or injury inflicted by a person on his or her own body in a repeated pattern, usually with a low risk of fatality and without deliberate suicidal intent"	Mental health services; all people who had self harmed but had recoivered or were in the process of recovering	Mental health professionals; therapists (including CBT); inpatient nurse;	UK	6	23–54	all women	burning, cutting, drinking harmful substances, trying to break bones, opinching and biting skin	The recovering self: Inconceivability of recovered self vs. recovery as a processChanges in internal sense of selfAccepting the selfRebuilding a new selfStriving for hopeEvolving relationship with self injury‐Striving for InsightParadoxical relationship with self‐injuryOwnership and self managementinlcusion v isolation‐Presence and absence of support in recoveryPositive and negative experiences of mental health services	3 (adequate)

The included studies provided 67 relevant quotations that were included in the meta‐analysis; we were unable to determine the number of individuals this represented, as not all quotations used participant labels or pseudonyms. The quality of the studies was generally high, with 70% of included studies rated as high quality. The breakdown of quotations under the overarching meta‐themes is presented in Table 4.

**Table 4:** Meta‐themes and sub‐headings in the meta‐synthesis

Positive aspects of seeing a professional	Not feeling alone
	Talking helps
Positive attributes of individual professionals	Reciprocating trust
	Genuine empathic concern
	Someone understanding self‐harm and the individual
	Non‐judgemental

Of the quotations containing references to therapy or counselling, none indicated the specific treatment approaches underpinning the interventions. Instead, direct accounts explored the beneficial aspects of talking to professionals in general, and the positive characteristics of individual therapists that were found to be helpful.

### Positive aspects of professional support

Direct accounts focused on positive interactions with a variety of professionals, including therapists and clinicians working in community and inpatient settings. Professionals could encourage positive thinking and help individuals to understand that they were not alone in their recoveries, to understand the potential benefits of reaching out to others in difficult situations, and to feel valued by people around them.

#### Not feeling alone

Many participants were reluctant to rely on social networks for support, whether concerned about damaging their existing relationships or unsure about the degree to which others would be willing to help them. Through contact with professionals, participants acknowledged a sense of comfort derived from knowing that help was reliably available:

Thanks to the telephone coaching you never feel left all by yourself, when a crisis comes along… It’s about getting support to get in to the right tracks of thinking, so you can use the skills to handle it yourself. (Perseius *et al*. 2003)

Participants discussed the benefits of working with professionals with the knowledge and skills to guide them through difficult situations. This enabled them to focus and gain confidence in responding to personal crises, within the safety of a therapeutic partnership. Professionals could find ways to remind those who self‐harmed that they experienced similar thoughts and feelings to other people, in the absence of peer networks; this was described as both normalizing and validating for feelings connected to self‐harm.

In order to receive this degree of help, participants acknowledged that they needed to meet professionals halfway in their efforts to share and engage with them.

#### Talking helps

Participants acknowledged the utility of talking to an individual in a professional role, with training and experience. An assumed degree of experience and competency in those with designated roles may have helped individuals to feel comfortable.

Some individuals found that once the initial step to reach out to a professional had been made, they felt comfortable discussing a broader range of issues that were causing mental distress, rather than focusing on the physical act of self‐harm. This could have made a positive impact on their overall mental wellbeing.

Talking openly to professionals could also help individuals become accustomed to opening up in personal relationships outside of therapy. In doing so, the availability of vital social support in times of crisis could increase beyond a single professional support:

I knew that talking would be helpful but it takes time before you are used to discussing openly all kinds of things with a psychologist or therapist. Now I have been in therapy for 2 years. Although my mother did not understand how bad I felt, chatting with her was enough to keep me from self‐mutilating that night. (Rissanen *et al*. 2009)

### Positive attributes of individual professionals

Direct accounts related to interactions with a variety of professionals, including therapists and clinicians working in community and inpatient settings.

#### Reciprocating trust

Participants in the included studies referred to the trust conveyed by experienced professionals, described by participants as ‘sincere… open’, and ‘always upfront and… completely consistent’ (Bergmans *et al*. 2009). Once individuals learned that someone believed in their ability to enact necessary changes, they were encouraged to adopt this belief in themselves:

I finally found a person [trauma therapist] who was able to connect with me and help me climb out of the pit I was in. I asked her if anyone as sick as me could get better, and she said, ‘Yes’, she had seen it happen. Those were the words I had been waiting to hear… It felt like she had become my lifeline to a better place outside of hospitals and as long as I knew someone was there for me, I didn’t have to die. (Chan *et al*. 2017)

If barriers to trusting professionals were not addressed, people seeking help for self‐harm may have felt unable to fully share their thoughts and feelings with their therapists in the relatively short time of the therapeutic contact.

In terms of reciprocity in therapy, participants appreciated professionals taking an active role in interventions, such as giving feedback and being clear about their reactions to expressed thoughts and beliefs:

Because of his (CAMHS counsellor) attitude, it kind of made me realize that it wasn’t necessarily talking to a stranger about my problems, it was talking to someone who could help, and that’s the difference. (McAndrew & Warne 2014)

Trust was something that could be cultivated and reciprocated; by learning to trust professionals and realizing the opportunities this created, a person could be motivated to trust others within their social networks.

#### Genuine empathic concern

Some of the direct accounts in the included studies reflected on professionals who conveyed genuine emotional connections with their clients. This was motivated by professionals who listened, without pushing ideals or advice onto them, and gave authentic feedback in their interactions:

I really had that sense that she honestly did care about me and how I was doing and she genuinely did want to help me and didn’t mind listening to me when I did talk and if I was blabbing on about something she just sat there and listened. (Bostik & Everall 2007)

First‐hand accounts described feeling warmth and compassion during their interactions with professionals and being treated with kindness. The professionals in these cases showed genuine human emotion and concern for the person’s welfare, serving as a reminder that they mattered:

He [general practitioner] was like rock. He really was, he was genuinely concerned for me and I could tell he was. He was really worried and in a way he made me feel better … You know that someone cared and he, you know, he would see me every, maybe every month every two months just to see how everything was and till he retired really so he was a great help. (Sinclair & Green 2005)

#### Someone understanding self‐harm and the individual

Professionals avoided making dismissive or inaccurate assumptions related to self‐harm and reflected genuine attempts to listen to and understand the person and how they were feeling at critical points. The direct accounts suggested that people appreciated moments where professionals would interact with them appropriately around their self‐harm, physically acknowledging evidence that a person had engaged in self‐harm and showing an interest in understanding the person and how self‐harm is a response to what is affecting them.

Being comfortable in discussing self‐harm extended to the therapist having an understanding of the challenges of interacting with services. Some professionals could intervene or advocate for them, easing the burden of managing an escalating crisis:

That nurse got to know me well after some time – managed to see when I became irritable… She sees from my body language that as it goes on now, I was beginning to be very angry. And the result then was that she took control of the conversation and said that we can talk about this; …And she then took the doctor aside, and later they came back and said that you will get the leave. (Vatne & Naden 2018)

Professionals could offer individuals a ‘safe space’ to express themselves freely, depending on the ability of professionals to accept the presence of disturbing thoughts and feelings.

The extent to which a professional can empathize with another person’s social or cultural context of their situation could help them to articulate relevant experiences and feel better understood during counselling:

…So during the one‐and‐a‐half‐hour counselling session, I spent more than a half of the session crying. I cried so much but after I cried, I felt better. Also because he was an immigrant himself too, he understood what I was going through. (Han & Oliffe 2015)

#### Non‐judgemental

The notion that others were willing to understand without judging the individual’s reasons for engaging in self‐harm was described by some as liberating.

Participants appreciated professionals who did not apply preconceived assumptions. These were a welcome relief for those who had endured unfair judgement from professionals because of their diagnosis or received marginalizing labels to describe their character:

I had a… caseworker… when they’re choosing a person to manage my case, they were basically drawing straws… like, “another unmotivated borderline. You have her” … she’s like… “I told them. … Why don’t I… meet her before you go telling me that she’s unmotivated.” … we really got along after a while, because she… pushed me to do well and she believed that I could. Where other people may treat you as if you’re chronic. Then you’ll act chronic. (Shaw 2006)

## DISCUSSION

The direct accounts featured in this literature review have demonstrated the key features of professional actions considered to serve an instrumental role in helping individuals to stop or reduce self‐harm. Our previous review highlighted the importance of interpersonal relationships in reduction of self‐harm (Brennan *et al*., in press), a concept mirrored in our findings about interactions with professionals. The presence of mutual trust, an empathic understanding of the person’s condition, and a willingness to help individuals based on their unique relationship with self‐harm and the outcomes they will hope to achieve throughout, were the professional attributes considered necessary to promote such a relationship, whether very brief (e.g., during a one–off contact in hospital) or sustained (such as with a therapist). It was important that such interactions were devoid of pre‐conceived judgements and assumptions. This is in line with findings taken from counselling practitioners working with people who self‐harm (Long & Jenkins 2010) and accounts from people experiencing thoughts of suicide, with supportive and caring interactions with professionals cited as the crux of therapy (Lakeman & FitzGerald 2008).

Very few of the people interviewed referred to specific techniques or strategies they had learned in therapy or in talking with a professional, and it was impossible from the accounts to determine what kinds of therapy people had received. This may be indicative of the priority individuals placed on their relationship with professionals, relative to modality of therapy or specific approaches. Direct accounts also reflected an appreciation for the structure gained through working with a therapeutic service. This provided a sense of needed stability and reliability during times of distress. Essential to these feelings of mutual trust was the perception that practitioners made no assumptions or judgements about them based on their diagnosis, or past experiences (Brown & Kimball 2013). Self‐harm may be accompanied by heightened feelings of internalized stigma and shame, and negative practitioner attitudes towards self‐harm may compound such feelings, putting certain aspects of the therapeutic relationship in jeopardy and therefore impede therapeutic outcomes (Long & Jenkins 2010).

Features of interactions highlighted by people in the studies as being helpful are all aspects of the so‐called common factors of psychotherapy and refer to shared aspects of therapy as opposed to ingredients or techniques that are specific to a particular kind of treatment.

Common factors include goal consensus, empathy, alliance (the strength of the bond between therapist and client), positive regard, genuineness, cultural adaptations of treatment, and expectations (Wampold 2015). Many of these common factors have been shown to be predictive of outcome of psychological treatment regardless of the modality of therapy (Flückiger *et al*. 2018; Wampold & Imel 2015). For example, the alliance is the most researched of the common factors and a meta‐analysis of the effect of the alliance on outcome included ~295 studies involving over 30 000 patients and found the relationship between alliance and outcome to be equivalent to that of a Cohen’s *d* of 0.578 (Flückiger *et al*. 2018). Recent work has established that there is a synergy between collaborative qualities in the therapist–client relationship and early distress remediation; in other words, symptom improvement goes hand in hand with a closer, warmer therapeutic bond (Flückiger *et al*. 2020).

The verbatim accounts from this review are strongly supported by empirical evidence that many of the most helpful aspects of psychological help are based upon the fundamental building blocks common to all therapies. Regardless of technique, it is the warmth and empathy of the therapist and the close trusting nature of the relationship between client and therapist that are crucial for a positive helpful outcome. This may provide the benefits of therapeutic interactions within a ‘neutral’ setting before the individual is ready to reconnect with members of their social network (Lakeman & FitzGerald 2008).

These common factors were not identified in isolation from the content of therapy. Current recommendations for intervention after self‐harm are for therapy focused on the problems that precede self‐harm and understanding the act and its meaning, and participants clearly valued therapists who were comfortable with discussing self‐harm in itself and the reasons for it. Unfortunately, criticism of health services response to self‐harm often focuses on an approach that is excessively preoccupied with risk assessment and brief contact, which need not be incompatible with the positive features discussed here but are often experienced as such.

### Strengths and limitations

The results of this review were limited to articles with an English‐language version; therefore, some accounts from different countries and culturally varied experiences of self‐harm may not have been represented. We were also bound by the quotation selection processes adopted by the original authors of the included articles. Some articles left gaps in contextual information, limiting the extent to which inferences could be made in relation to the nature and context of the professional interactions discussed. However, taking a broad definition of professional contact allowed us to explore the universally valued qualities of professionals from a variety of disciplines involved in providing help for self‐harm, allowing observations to be made across general practice, inpatient care and psychological therapy. This review also supported the promotion of a person‐centred understanding of self‐harm through a focus on first‐hand accounts, thus amplifying the voices of those with personal experience (Brennan *et al*., in press).

The findings of this review have indicated the importance of non‐specific components of therapy to facilitate a reduction or cessation of self‐harm. Therapist attributes necessary for such an experience in therapy may be facilitated in the future by improved training and education for all professionals likely to be involved in helping people who self‐harm. This should be coupled with improved access to timely and reliable help which validates the person’s feelings and fulfils their need for safety and understanding (Warm *et al*. 2002). The need to combine such common factors with the delivery of large‐scale problem‐oriented help to the diverse population of people who self‐harm is a challenge for current mental health services in the UK. Further accounts of people’s experiences of therapy delivered in a clinical setting, for example, IAPT or specific mental health services, would help to shape the current treatments available, including their duration and intensity, and the emphasis they place on common factors such as alliance and empathy.

## RELEVANCE TO CLINICAL PRACTICE

The findings of this review indicate that the therapeutic alliance with professionals is considered a crucial component of therapy by people who have reduced or stopped self‐harm, regardless of the underpinning philosophy. Such relationships should be more accessible and timely for all those who self‐harm, via the provision of appropriate and person‐centred support.

## Funding information

This study/project is funded by the National Institute for Health Research (NIHR) Programme Grants for Applied Research. The views expressed are those of the author(s) and not necessarily those of the NIHR or the Department of Health and Social Care.

## Supporting information

Supplementary Material
