# Supplementary material for: Valued attributes of professional support for people who repeatedly self‐harm: A systematic review and meta‐synthesis of first‐hand accounts
Source: Int J Ment Health Nurs. 2022 Jan 15;31(2):424–41. doi: 10.1111/inm.12969 (PMC9306637; doi:10.1111/inm.12969)
Supplement: Supplementary file 1 — Supplementary Material [file INM-31-424-s001.doc]

Database: Ovid MEDLINE(R) <1946 to November Week 4 2018>

Search Strategy:

--------------------------------------------------------------------------------

1 self-injurious behavior/ (6824)

2 self mutilation/ (3168)

3 Suicidal Ideation/ or Suicide, Attempted/ or suicide/ (52849)

4 (themsel* adj2 (aggress* or burn* or scratch* or bit* or intoxicat* or harm* or cutt* or cut? or immolat* or inflict* or injur* or mutilat* or poison* or damag* or destruct*)).tw,kf. (859)

5 (oneself adj2 (aggress* or burn* or scratch* or bit* or intoxicat* or harm* or cutt* or cut? or immolat* or inflict* or injur* or mutilat* or poison* or damag* or destruct*)).tw,kf. (80)

6 (self adj2 (aggress* or burn* or scratch* or bit* or intoxicat* or harm* or cutt* or cut? or immolat* or inflict* or injur* or mutilat* or poison* or damag* or destruct*)).tw,kf. (15383)

7 (parasuicid* or para-suicid*).tw,kf. (616)

8 (autoaggress* or automutilat*).tw,kf. (843)

9 (auto adj (aggress* or mutilat* or destructi*)).tw,kf. (252)

10 (poison adj2 (deliberat* or intentional or intended)).tw,kf. (10)

11 (overdos* adj2 (deliberat* or intentional or intended)).tw,kf. (432)

12 suicidality.tw,kf. (4358)

13 (suicid* not (assisted adj suicide?)).tw,kf. (59151)

14 NSSI.tw,kf. (616)

15 DHS.tw,kf. (2304)

16 (selfharm* or selfinjur* or selfinflict*).tw,kf. (17)

17 (headbang* or head-bang*).tw,kf. (156)

18 or/1-17 [Self Harm] (89266)

19 Harm Reduction/ (2532)

20 Risk Reduction Behavior/ (11037)

21 [term removed before actual search]

22 Avoidance Learning/ (21274)

23 Self control/ (1447)

24 (Stop* or remitt* or resoluti* or "no longer" or remissi* or suspend* or reduc* or overcom* or abstain* or "giv* up*" or cessat* or minimi* or coping or cope or "self control" or "protect* against" or "protect* effect*" or (regulation adj3 (strateg* or skill* or abilit* or emotional or style?))).ti,kf. (421871)

25 (moderat* or diminution* or mitigat* or minimis* or avoid* or ceas* or cess* or discontin* or refrain* or supress* or decreas* or diminish* or mitigat* or lessen* or shorten* or inhibit* or recover* or prevent*).ti,kf. (1004107)

26 ((turn* or keep* or cut* or slim* or bring*) adj4 down).ti,kf. (592)

27 (behavio?r* and (chang* or modif* or alter* or reshap* or shift* or improv* or transform* or swap* or swop*)).ti,kf. (22405)

28 ((Stop* or remitt* or resoluti* or "no longer" or remission* or suspend* or reduc* or overcom* or abstain* or "giv* up*" or cessat* or minimi* or coping or cope or "self control" or protect*) adj4 (Technique* or factor* or strateg* or skill* or abilit * or style? or effect* or mechanism* or way? or tip? or behaviour* or behavior* or function*)).ab. (475542)

29 ((moderat* or diminution* or mitigat* or minimis* or avoid* or ceas* or cess* or discontin* or refrain* or supress* or decreas* or diminish* or mitigat* or lessen* or shorten* or inhibit* or recover* or prevent*) adj4 (Technique* or factor* or strateg* or skill* or abilit * or style? or effect* or mechanism* or way? or tip? or behaviour* or behavior* or function*)).ab. (743158)

30 ((turn* or keep* or cut* or slim* or bring*) adj4 down adj4 (Technique* or factor* or strateg* or skill* or abilit * or style? or effect* or mechanism* or way? or tip? or behaviour* or behavior* or function*)).ab. (240)

31 (behavio?r* adj4 (chang* or modif* or alter* or reshap* or shift* or improv* or transform* or swap* or swop*)).ab. (84654)

32 exp Adaptation, Psychological/ (119279)

33 Resilience, Psychological/ (4137)

34 Choice behavior/ (29908)

35 (Stop* or remitt* or resoluti* or "no longer" or remissi* or suspend* or reduc* or overcom* or abstain* or "giv* up*" or cessat* or minimi* or coping or "self control" or protect* or (regulation adj3 (strateg* or skill* or abilit* or emotional or style?)) or moderat* or diminution* or mitigat* or minimis* or avoid* or ceas* or cess* or discontin* or refrain* or supress* or decreas* or diminish* or mitigat* or lessen* or shorten* or inhibit* or recover* or prevent* or (behavio?r* adj4 (chang* or modif* or alter* or reshap* or shift* or improv* or transform* or swap* or swop*))).tw,kf. (7639262)

36 (32 or 33 or 34) and 35 (62065)

37 or/19-31,36 [Reduction Strategies] (2359284)

38 and/18,37 [Self Harm +Reduction] (13064)

39 ((Stop* or remitt* or resoluti* or "no longer" or remissi* or suspend* or reduc* or overcom* or abstain* or "giv* up*" or cessat* or minimi* or coping or "self control" or "protect* against" or "protect* effect*" or (regulation adj3 (strateg* or skill* or abilit* or emotional or style?))) adj2 (suicid* or ((self* or onesel* or themsel*) adj2 (aggress* or burn* or scratch* or bit* or intoxicat* or harm* or cutt* or cut? or immolat* or inflict* or injur* or mutilat* or poison* or damag* or destruct*)))).ab. (1591)

40 ((moderat* or mitigat* or minimis* or avoid* or ceas* or cess* or discontin* or refrain* or supress* or decreas* or diminish* or mitigat* or lessen* or shorten* or prevent*) adj2 (suicid* or ((self* or onesel* or themsel*) adj2 (aggress* or burn* or scratch* or bit* or intoxicat* or harm* or cutt* or cut? or immolat* or inflict* or injur* or mutilat* or poison* or damag* or destruct*)))).ab. (4554)

41 ((turn* or keep* or cut* or slim* or bring*) adj4 down adj4 (suicid* or ((self* or onesel* or themsel*) adj4 (aggress* or burn* or scratch* or bit* or intoxicat* or harm* or cutt* or cut? or immolat* or inflict* or injur* or mutilat* or poison* or damag* or destruct*)))).ab. (11)

42 (behavio?r* adj4 (chang* or modif* or alter* or reshap* or shift* or improv* or transform* or swap* or swop*) adj4 (suicid* or ((self* or onesel* or themsel*) adj4 (aggress* or burn* or scratch* or bit* or intoxicat* or harm* or cutt* or cut? or immolat* or inflict* or injur* or mutilat* or poison* or damag* or destruct*)))).ab. (143)

43 or/38-42 [Self Harm reduction] (15880)

44 exp qualitative research/ (42526)

45 "Surveys and Questionnaires"/ (411589)

46 interview/ (27560)

47 Interviews as Topic/ (55630)

48 Narration/ (7336)

49 qualitative.tw,kf. (160720)

50 questionnaire*.tw,kf. (383976)

51 ethnological research.tw,kf. (7)

52 ethnograph*.tw,kf. (7958)

53 ethnonursing.tw,kf. (103)

54 phenomenol*.tw,kf. (17628)

55 (grounded adj (theor$ or study or studies or research or analys?s)).tw,kf. (8563)

56 (focus adj group*).tw,kf. (31704)

57 Self Report/ (24725)

58 (Self-report* or "First hand").tw,kf. (114543)

59 ((patient? or inpatient? or outpatient? or client? or young* or youth* or teenag* or adolescent* or adult* or m?n or wom?n or girl? or boy? or people or person* or attempter? or lived or participant? or sufferer?) adj4 (view* or perspective* or insight* or vision* or account? or report* or Testimon* or Experience? or Narrati* or Opinion* or Stories or Story or Storytelling or Voice*)).tw,kf. (541630)

60 or/44-59 [Qualitative Filter] (1304945)

61 and/43,60 [Self Harm reduction + Qualitative Filter] (3641)

***************************

<1>

Unique Identifier

28758704

Title

An Ecological Investigation of the Emotional Context Surrounding Nonsuicidal Self-Injurious Thoughts and Behaviors in Adolescents and Young Adults.

Source

Suicide & Life-Threatening Behavior. 48(2):149-159, 2018 04.

Authors

Kranzler A; Fehling KB; Lindqvist J; Brillante J; Yuan F; Gao X; Miller AL; Selby EA.

Authors Full Name

Kranzler, Amy; Fehling, Kara B; Lindqvist, Janne; Brillante, Julia; Yuan, Fengpeng; Gao, Xianyi; Miller, Alec L; Selby, Edward A.

Publication Type

Journal Article. Research Support, Non-U.S. Gov't. Research Support, U.S. Gov't, Non-P.H.S..

Link to the Ovid Full Text or citation:

[Click here for full text options](http://ovidsp.ovid.com/ovidweb.cgi?T=JS&CSC=Y&NEWS=N&PAGE=fulltext&D=medl&AN=28758704)
